# A Novel Approach to Identifying Physical Markers of Cryo-Damage in Bull Spermatozoa

**DOI:** 10.1371/journal.pone.0126232

**Published:** 2015-05-04

**Authors:** Sung-Jae Yoon, Woo-Sung Kwon, Md Saidur Rahman, June-Sub Lee, Myung-Geol Pang

**Affiliations:** Department of Animal Science and Technology, Chung-Ang University, Anseong, Gyeonggi-do, 456–756, Republic of Korea; University of Nevada School of Medicine, UNITED STATES

## Abstract

Cryopreservation is an efficient way to store spermatozoa and plays a critical role in the livestock industry as well as in clinical practice. During cryopreservation, cryo-stress causes substantial damage to spermatozoa. In present study, the effects of cryo-stress at various cryopreservation steps, such as dilution / cooling, adding cryoprtectant, and freezing were studied in spermatozoa collected from 9 individual bull testes. The motility (%), motion kinematics, capacitation status, mitochondrial activity, and viability of bovine spermatozoa at each step of the cryopreservation process were assessed using computer-assisted sperm analysis, Hoechst 33258/chlortetracycline fluorescence, rhodamine 123 staining, and hypo-osmotic swelling test, respectively. The results demonstrate that the cryopreservation steps reduced motility (%), rapid speed (%), and mitochondrial activity, whereas medium/slow speed (%), and the acrosome reaction were increased (P < 0.05). Differences (Δ) of the acrosome reaction were higher in dilution/cooling step (P < 0.05), whereas differences (Δ) of motility, rapid speed, and non-progressive motility were higher in cryoprotectant and freezing as compared to dilution/cooling (P < 0.05). On the other hand, differences (Δ) of mitochondrial activity, viability, and progressive motility were higher in freezing step (P < 0.05) while the difference (Δ) of the acrosome reaction was higher in dilution/cooling (P < 0.05). Based on these results, we propose that freezing / thawing steps are the most critical in cryopreservation and may provide a logical ground of understanding on the cryo-damage. Moreover, these sperm parameters might be used as physical markers of sperm cryo-damage.

## Introduction

Sperm cryopreservation is an important means for assisted reproductive technique and is the most efficient way for storing genetic resources [1, 2]. Cryopreservation plays a major role in genetic improvement, economization of breeding programs in the livestock industry, and preservation of endangered species, and is clinically valuable in the management of infertility [3–6]. Cryopreservation has been applied to various species, including humans, swine, cattle, cats, and dogs, among others [2]. Although the goal of sperm cryopreservation is to preserve sperm motility, metabolic function, and fertility, the freeze—thawing process inevitably causes damage to spermatozoa, thereby reducing fertility [3, 4, 7, 8].

Cryopreservation consists of three steps—dilution with the extender/cooling, addition of cryoprotectant (CP), and freeze—thawing [4, 9]—during which spermatozoa are subjected to various stresses such as cold shock, osmotic and oxidative stress, and intracellular ice crystal formation [1, 9, 10]. These cause damage to sperm integrity, membrane structure, and sperm function [6, 9, 11, 12]. In addition, excessive mitochondrial activity induces the generation of reactive oxygen species that affect cellular compounds and organelle functions [13]. Furthermore, temperature-sensitive membranes and cytoskeletal structures, ultimately resulting in reduced sperm motility, viability, and fertility [14].

Cryopreservation protocols have been developed and optimized over the last several decades. Previous studies have provided evidences that freezing-thawing affects sperm functions and semen quality [12, 15]. Several studies have compared spermatozoa before and after cryopreservation [7, 12, 16, 17] or attempted to identify the mechanisms underlying specific steps of the process [18–21]. However, there have been no comprehensive studies examining each step of cryopreservation in relation to functional parameters of spermatozoa. Therefore, we attempted to investigate the effects of the different steps of cryopreservation on bovine spermatozoa by evaluating motility, motion kinematics, viability, capacitation status, and mitochondrial activity.

## Materials and Methods

### Ethical statement

All animal procedures were performed in accordance with the guidelines for the ethical treatment of animals, and were approved by the Institutional Animal Care and Use Committee of Chung-Ang University, Seoul, Korea.

### Sample collection

Native Korean Bull (Hanwoo) testes were obtained from a local slaughterhouse (Seomun Co., Hwaseong, Korea) and transferred to the laboratory within 3 h on ice [22]. Sperm samples were collected from nine individual bull epididymides. A small cut was made at the cauda epididymidis of the testis avoiding vessel. Phosphate-buffered saline (PBS; Sigma-Aldrich, St Louis, MO, USA) was backflushed into the end of the vas deferens using a 10-ml syringe. Flushed samples were washed at 700 × *g* for 15 min [23–25].

### Cryopreservation of spermatozoa

Sperm cryopreservation was performed as previously described [26]. Briefly, washed samples (Control) were diluted (100 × 10^6^ cells/ml) in Tris—egg yolk buffer (TYB; 250 mM Tris, 88.5 mM citric acid, 68.8 mM glucose, and 20% egg yolk) and cooled to 4°C over 2 h (Step 1). An equal volume of TYB with 12% glycerol was added to extend the sample, which was then equilibrated at 4°C for 2 h (Step 2). Equilibrated samples were packaged into 0.5-ml straws and frozen in liquid nitrogen vapor 2.5cm above the liquid nitrogen for 15 min, and then plunged into liquid nitrogen for storage. Straws were thawed at 37°C for 1 min after 2 weeks of cryopreservation (Step 3).

### Computer-assisted sperm analysis (CASA)

Sperm motility (%) and kinematics at each step of cryopreservation were analyzed using a CASA system (ISAS ver.1.2; Valencia, Spain). Briefly, 10 μl of sample were placed in a 37°C Makler chamber (Makler, Haifa, Israel). Using the 10× objective in-phase contrast mode, the image was relayed, digitized, and analyzed with the ISAS software. The movement of at least 250 sperm cells was recorded for each sample from more than five randomly selected fields per replicate, and used to analyze sperm motility (%), kinematics (progressively or non-progressively motile), and speed parameters (rapid, > 50 μm/s; medium, 25–50 μm/s; and slow, < 25 μm/s).

### Assessment of capacitation status by Hoechst 33258 (H33258)/chlortetracycline fluorescence (CTC)

Capacitation status was assessed by the H33258/CTC dual staining method as previously described [27, 28]. Briefly, 15 μl of H33258 solution (10 μg H33258/ml PBS) were added to 135 μl of sample and the mixture was incubated for 10 min at room temperature; 250 μl of 2% (w/v) polyvinylpyrrolidone (Sigma-Aldrich) in PBS were then added to remove excess dye, followed by centrifugation at 700 × *g* for 5 min. The supernatant was discarded and the pellet was resuspended in 100 μl of PBS and 100 μl of CTC solution (750 mM CTC in 5 μl buffer composed of 20 mM Tris, 130 mM NaCl, and 5 mM cysteine, pH 7.4). Capacitation status was assessed using a Microphot-FXA microscope (Nikon, Tokyo, Japan) under epifluorescent illumination using ultraviolet BP 340–380/LP 425 and BP 450–490/LP 515 excitation/emission filters for H33258 and CTC, respectively. Spermatozoa capacitation patterns were classified as live non-capacitated (F; green fluorescence distributed uniformly over the entire sperm head, with or without a stronger fluorescent line at the equatorial segment), live capacitated (B; green fluorescence over the acrosomal region and a dark post-acrosomal region), or live acrosome-reacted (AR; sperm showing mottled green fluorescence over the head, green fluorescence only in the post-acrosomal region, or no fluorescence over the head). Capacitation status was evaluated in at least 400 spermatozoa per slide.

### Hypoosmotic swelling test (HOST)

Sperm viability and the functional integrity of the membrane were determined by the HOST as previously described [29]. Briefly, 900 μl of hypo-osmotic solution (0.9% NaCl in distilled water, 150 mOsm/kg) were added to 100 μl of sample and incubated for 30 min at 37°C in an atmosphere of 5% CO_2_. A drop of sample was smeared on a clean slide, allowed to air dry, and fixed with freshly prepared fixative solution. Membrane swelling patterns were evaluated using Microphot-FXA microscope (Nikon). HOST was carried out in at least 400 spermatozoa per slide.

### Mitochondrial activity

Mitochondrial membrane potential was measured by rhodamine 123 (R123; Sigma-Aldrich) staining as previously described [30, 31]. Briefly, 15 μl of R123 (0.01 mg R123/ml PBS) were added to 100 μl of sample and incubated for 15 min at 37°C in an atmosphere of 5% CO_2_. After incubation, the sample was centrifuged at 700 × *g* for 5 min and resuspended in 1 ml PBS. Samples were analyzed by flow cytometry (Becton Dickinson, Franklin Lakes, NJ, USA) with excitation and emission wavelengths of 488 and 525 nm, respectively. A total of 1 0,000 cells in each sample were analyzed with CellQuest software (Becton Dickinson).

### Statistical analysis

Numerical values were obtained from different sperm parameters (such as, motility, motion kinematics, capacitation status, viability, mitochondrial activity) in each 9 individual samples. Treatment group was classified as Control (Fresh sperm), Step 1 (Dilution / Cooling), Step 2 (Adding cryoprotectant) and Step 3 (Freezing / Thawing). Data were analyzed with SPSS v.21.0 (SPSS Inc., Chicago, IL, USA). One-way analysis of variance was used to compare values of 4 treatment groups in sperm parameters and values of differences (△) of sperm parameters in each cryopreservation steps with Tukey’s test to detect differences. Correlation among different parameters in each steps of cryopreservation was performed by pearson correlation test. *P* < 0.05 was considered statistically significant. Data are expressed as mean ± SEM.

## Results

**Fig 1:**
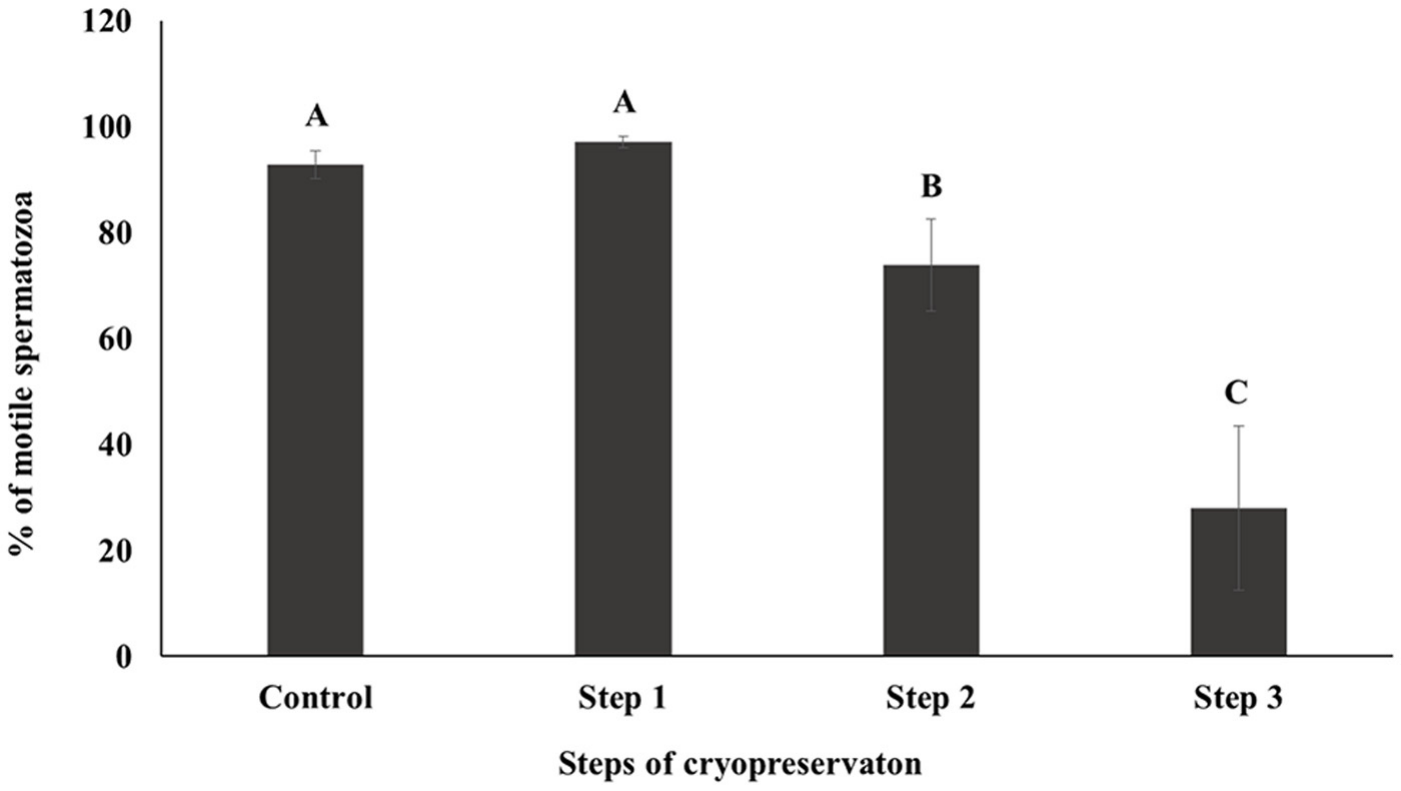
Sperm motility was determined by CASA. Control; fresh sperm, Step 1; dilution / cooling, Step 2; adding cryoprotectant, Step 3; freezing / thawing. Data are presented as the mean ± SE. Values with different superscripts (A, B, C) differed significantly between cryopreservation steps by one-way analysis of variance (P < 0.05, n = 9).

**Fig 2:**
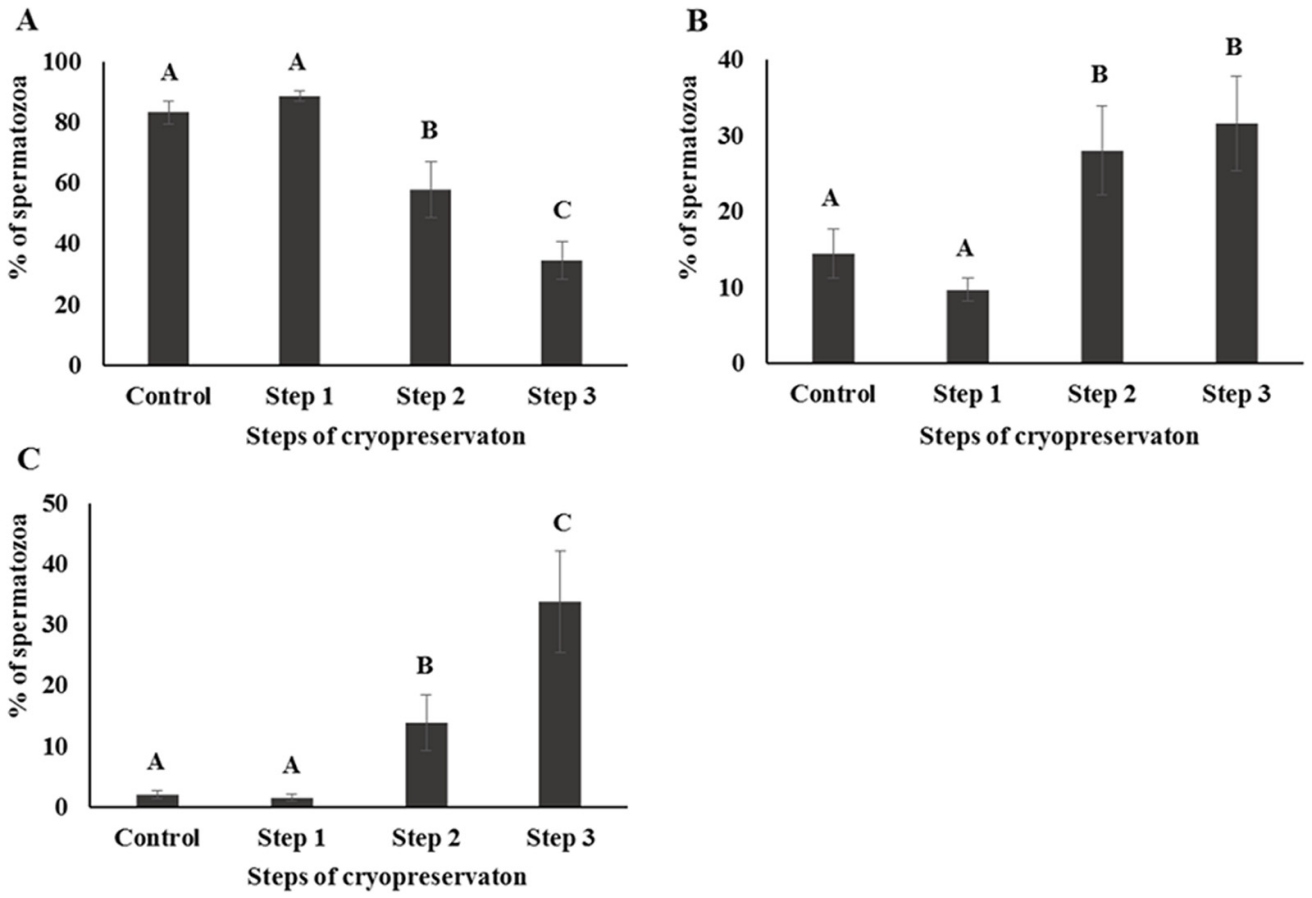
Sperm speed was determined by CASA and classified as (A) rapid (> 50 μm/s), (B) medium (25–50 μm/s), or (C) slow (< 25 μm/s). Control; fresh sperm, Step 1; dilution / cooling, Step 2; adding cryoprotectant, Step 3; freezing / thawing. Data are presented as mean ± SE. Values with different superscripts (A, B, C) differed significantly between cryopreservation steps by one-way analysis of variance (*P* < 0.05, n = 9).

**Fig 3:**
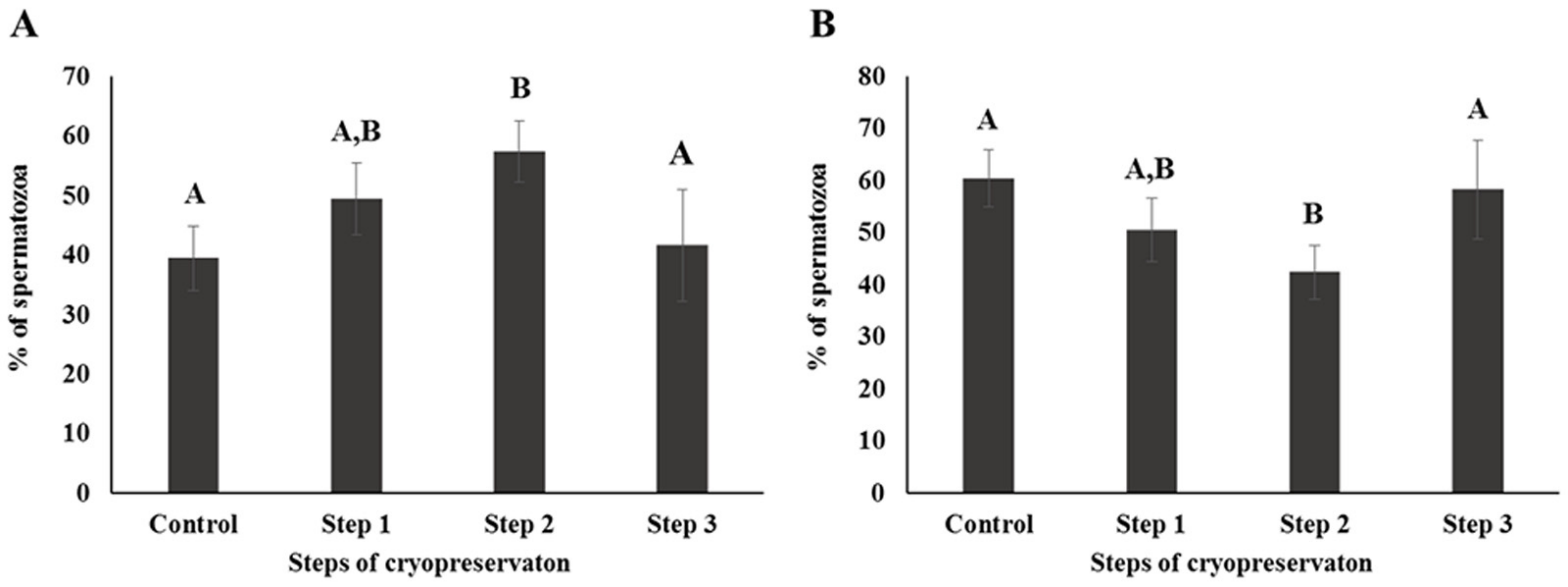
Sperm movement was determined by CASA and classified as (A) progressively motile or (B) non-progressively motile. Control; fresh sperm, Step 1; dilution / cooling, Step 2; adding cryoprotectant, Step 3; freezing / thawing. Data are presented as mean ± SE. Values with different superscripts (A,B,C) differed significantly between cryopreservation steps by one-way analysis of variance (*P* < 0.05, n = 9).

CASA was performed to investigate the motility and kinematics of spermatozoa at each step of cryopreservation. Motility and rapid speed were significantly decreased whereas medium and slow speed were significantly increased during cryopreservation. However, there were no differences between Control and Step 1 samples in terms of motility and motion kinematics (*P* < 0.05; Figs 1 and 2). Progressive motility was significantly higher and non-progressive motility was significantly lower in Step 2, whereas no differences were detected between Control, Step 1, and Step 3 samples (*P* < 0.05; Fig 3).

**Fig 4:**
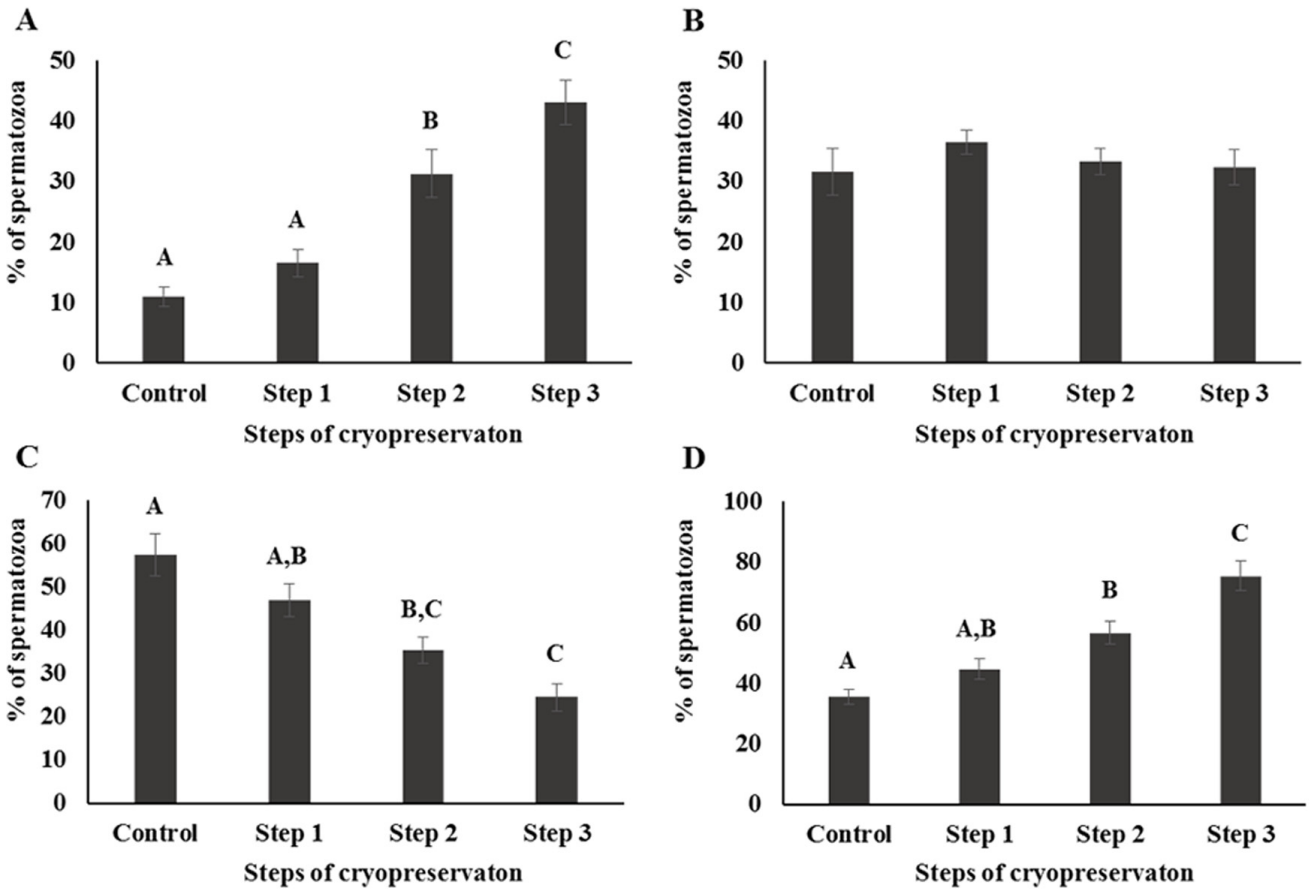
Capacitation patterns were assessed by the H33258/CTC dual staining method and classified as (A) live acrosome-reacted (AR), (B) live capacitated (B), (C) live non-capacitated (F), or (D) dead (D). Control; fresh sperm, Step 1; dilution / cooling, Step 2; adding cryoprotectant, Step 3; freezing / thawing. Data are presented as the mean ± SE. Values with different superscripts (A, B, C) differed significantly between cryopreservation steps by one-way analysis of variance (*P* < 0.05, n = 9).

**Fig 5:**
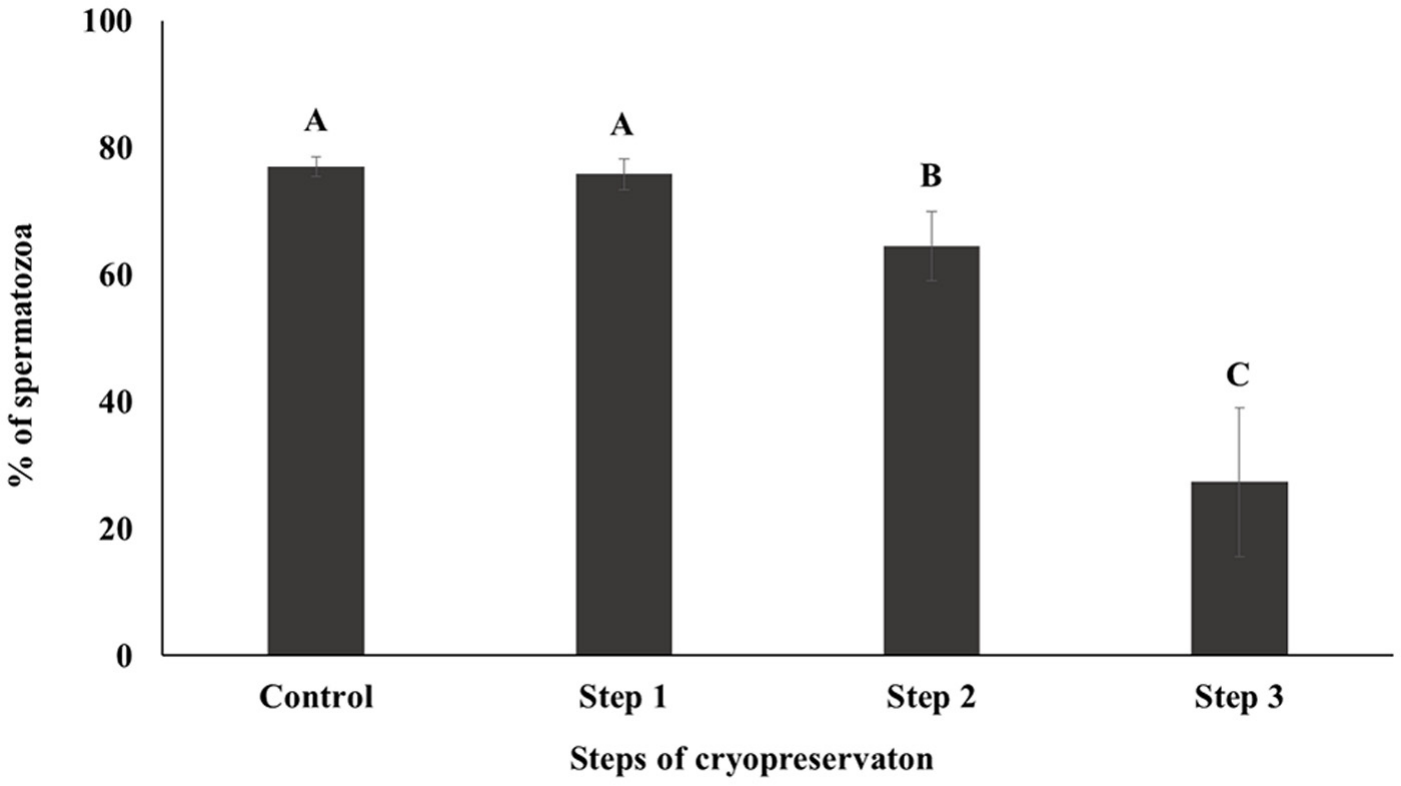
Mitochondrial activity was assessed by rhodamine 123 staining. Control; fresh sperm, Step 1; dilution / cooling, Step 2; adding cryoprotectant, Step 3; freezing / thawing. Data are presented as the mean ± SE. Values with different superscripts (A, B, C) differed significantly between cryopreservation steps by one-way analysis of variance (*P* < 0.05, n = 9).

**Fig 6:**
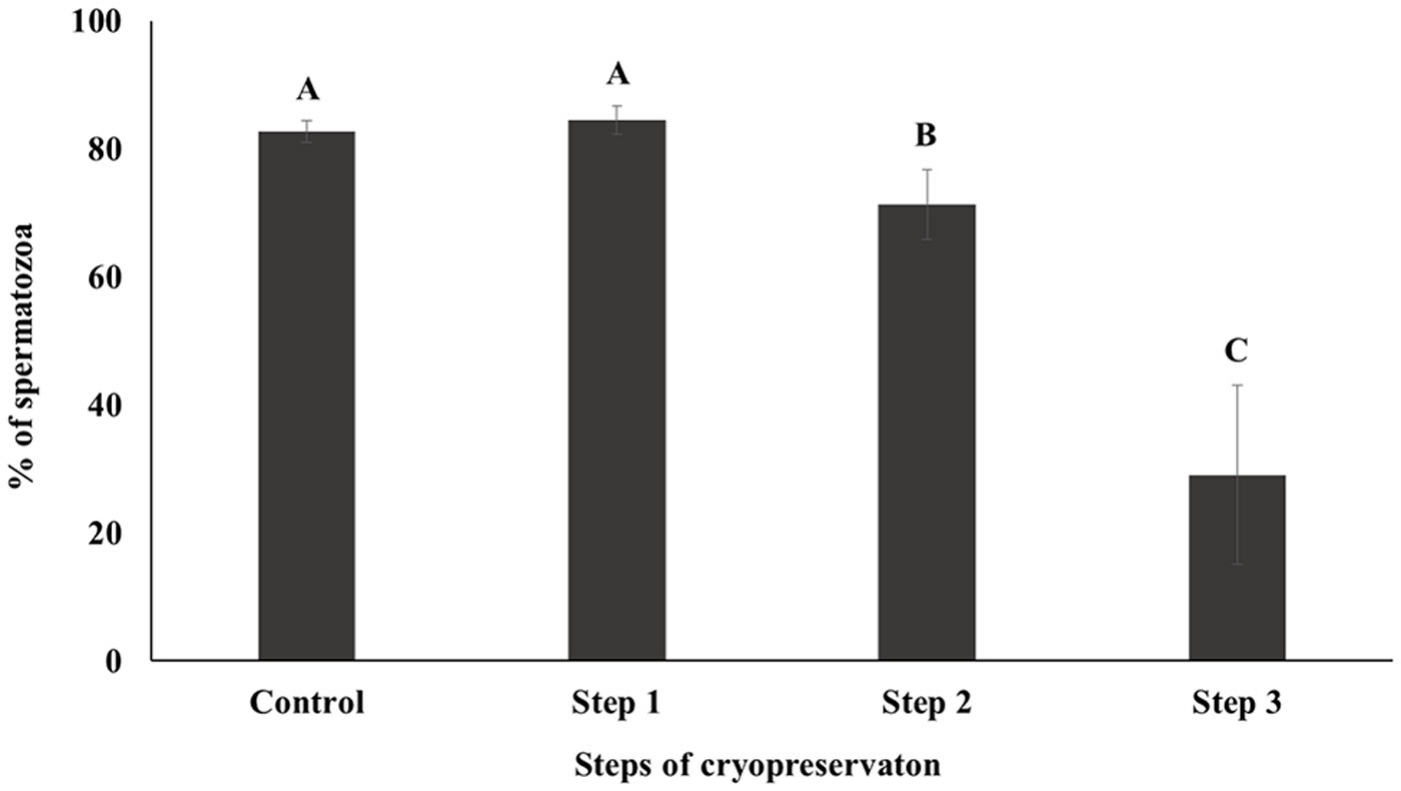
Sperm viability and the functional integrity of the membrane were determined by the HOST. Control; fresh sperm, Step 1; dilution / cooling, Step 2; adding cryoprotectant, Step 3; freezing / thawing. Data are presented as the mean ± SE. Values with different superscripts (A, B, C) differed significantly between cryopreservation steps by one-way analysis of variance (*P* < 0.05, n = 9).

Capacitation status was evaluated by H33258/CTC dual staining. The AR pattern was increased and F pattern was decreased (*P* < 0.05), while the B pattern remain unchanged (Fig 4). Mitochondrial activity was decreased during sperm cryopreservation, as determined by rhodamine 123 staining and flow cytometry (*P* < 0.05; Fig 5). HOST was performed to evaluate sperm viability and membrane function. The fraction of dead or membrane-damaged spermatozoa were increased during cryopreservation (*P* < 0.05; Fig 6). Differences (△) of AR and B patterns were higher in Step 1 than other parameters (*P* < 0.05; Table 1); the difference (△) of slow-speed sperm was highest for Steps 2 and 3 (*P* < 0.05; Table 1), as were differences (△) of motility, rapid speed, non-progressive motility of sperm (*P* < 0.05). Medium speed was higher in Step 1, whereas progressive motility, mitochondrial activity, and viability were higher in Step 3 (*P* < 0.05; Table 1). The difference (△) of slow speed was highest at every step of cryopreservation (*P* < 0.05, Table 1). Correlation among different parameters in each steps of cryopreservation were shown in supporting information (S1, S2, S3 and S4 Tables).

**Table 1:** Difference values of sperm parameters during cryopreservation.

Process of cryopreservation	Motility	Rapid speed	Medium speed	Slow speed	Progressively motile	Non-Progressively motile	AR pattern	B pattern	F pattern	D pattern	Mitochondrial activity	Viability
Control vs. Step 1	0.05±0.03^A,a^	0.07±0.04^A,a^	0.31±0.10^A,a,b,c,d^	0.46±0.15^A,b,c,d^	0.35±0.13^A,a,b,c,d^	0.17±0.09^A,a,b^	0.64±0.30^d^	0.58±0.55^c,d^	0.31±0.08^A,a,b,c,d^	0.26±0.11^A,a,b,c^	0.03±0.02^A,a^	0.04±0.02^A,a^
Step 1 vs. Step 2	0.25±0.10^B,a^	0.37±0.11^B,a^	1.43±0.49^B,a^	10.57±9.62^A,B,b^	0.41±0.19^A,a^	0.37±0.10^B,a^	0.77±0.33^a^	0.43±0.23^a^	0.29±0.10^A,a^	0.36±0.14^A,a^	0.15±0.06^A,a^	0.16±0.05^A,a^
Step 2 vs. Step 3	0.50±0.14^C,a^	0.28±0.06^B,a^	0.97±0.48^A,B,a^	15.61±8.02^A,B,b^	0.96±0.16^B,a^	0.40±0.09^B,a^	0.10±0.35^a^	0.56±0.34^a^	0.23±0.07^A,a^	0.57±0.26^A,a^	0.49±0.11^B,a^	0.52±0.13^B,a^
Control vs. Step 2	0.21±0.13^A,Ba^	0.31±0.14^B,a^	1.23±0.31^A,B,a^	10.49±1.37^A,B,b^	0.36±0.21^A,a^	0.45±0.09^B,a^	1.04±0.31^a^	0.47±0.24^A,a^	0.57±0.13^B,a^	0.62±0.19^A,a^	0.16±0.12^A,a^	0.14±0.11^A,a^
Control vs. Step 3	0.7±0.17^D,a^	0.58±0.12^C,a^	1.58±0.40^B,a^	25.77±1.77^B,b^	0.79±0.16^B,a^	0.85±0.12^C,a^	1.03±0.38^a^	0.6±0.17^A,a^	0.8±0.15^C,a^	1.19±0.26^B,a^	0.65±0.17^B,a^	0.65±0.18^B,a^

Data are presented as mean ± SE. Superscripts A, B, C, and D indicate significant differences between cryopreservation steps; a,b,c, and d indicate significant differences between sperm parameters by one-way analysis of variance (P < 0.05, n = 9).

## Discussion

During cryopreservation, spermatozoa are exposed to a stressful environment that includes cold shock, osmotic stress, and ice crystal formation [9, 12]. These stresses cause irreversible damage to sperm structure and function, resulting in a loss of up to 50% of viable spermatozoa [3]. It has been reported that methods of sperm cryopreservation for various species of domestic animals and humans are different, however basic principles of preservation are similar [32, 33]. Therefore, our study investigates the effects of the different steps of cryopreservation considering bull spermatozoa as a standard model. To evaluate the physical parameter most sensitive to cryo-damage, the cryopreservation steps were classified as dilution / cooling (Step 1), the addition of CP (Step 2), and freeze / thawing (Step 3) and sperm parameters were assessed at each step.

Motility is one of the most important factors in assessing sperm quality and essential for transporting the spermatozoa to the site of fertilization [34–36]. On the other hand, motion kinematics assessed by CASA provide an accurate representation of sperm movement objectively [37]. Our results showed that motility and rapid speed declined markedly during cryopreservation (Figs 1 and 2). The energy (ATP) used for sperm motility is mostly contributed by mitochondrial respiration [33]. Thus motility is closely related to mitochondrial activity [33, 38]. Consistent with these findings decrease in mitochondrial activity was also observed in current study during cryopreservation (Fig 5). Additionally, capacitation and the acrosome reaction are essential processes that allow spermatozoa to fertilize an oocyte [39, 40]. Spermatozoa are unable to fertilize an oocyte before capacitation, even they are motile and morphologically normal [41]. Simultaneously, structural and functional modifications occur during the acrosome reaction that required for sperm binding to zona pellucida [42, 43]. It has been reported that intracellular Ca2+ in spermatozoa is induced during cryopreservation and uptake of Ca2+ is a trigger of capacitation and the acrosome reaction [3, 44]. Therefore, these parameters are closely related to sperm viability and are key factors for determining semen quality / fertility [9, 12, 45, 46]. We demonstrated that slow speed / viability and mitochondrial activity / viability between Control and Step 1 are correlated significantly (S1 Table). Additionally, significant correlation was also observed among mitochondrial activity, motility, and viability (S2, S3, and S4 Tables). Moreover, AR pattern and slow speed were significantly correlated during cryopreservation (S4 Table). Therefore, taken together these findings suggest that various sperm parameters are associated each step of cryopreservation.

Step 1 of cryopreservation is associated with cold shock, which affects the physical properties and function of cell membranes [44, 47]. Membrane fluidity, intracellular potassium concentration, and tolerance to changes in osmolarity are all disrupted within the temperature range of 0–15°C [3, 9, 46]. However, there were no significant differences observed in the sperm parameters for Step 1 in this study (Figs 1–5). Interestingly, the difference of (△) AR was significantly higher in Step 1 than in other steps. It has been reported that membrane cytoskeletal components such as actin are sensitive to temperature, which can induce the depolymerization of actin filaments [3]; this may also related with acrosomal membrane of spermatozoa [48, 49]. It can therefore be supposed that the acrosomal membrane structure is most sensitive during the Step 1 (Table 1).The addition of CP causes osmotic stress, which is relevant to Step 2 as well to as thawing process during Step 3 [21, 45]. This can induce a change in the volume of spermatozoa due to an alteration in membrane permeability [3], causing damage to sperm surface proteins, and consequent loss of viability and reducing the capacity for fertilization [3, 9, 45]. CP also cause oxidative damage to sperm membrane phospholipids [50], resulting in the loss of motility, viability, and mitochondrial potential [51–53]. Furthermore, the toxicity of CPs can affect some components of the sperm membrane [54].

Step 3 of cryopreservation, which is associated with ice crystal formation, is typically accompanied by osmotic pressure changes [3, 9]. When a sample is cooled to below the freezing point, water crystallizes as ice; intra- and extracellular ice crystal formation causes damage to sperm membrane structures [44]. In addition, osmotic stress destroys spermatozoa during thawing [45, 55]. Accordingly, in the present study, Step 3 showed the highest differences (△) of various physical parameters including motility, slow speed, mitochondrial activity, and viability (Table 1).

Physical parameters were variably affected by cryo-damage at different steps of cryopreservation. In the present study, various parameters were assessed to identify physical markers at each step. In Step 1, differences (△) of AR and B patterns were significantly higher than in other steps. However, a difference (△) of slow speed was the only parameter that was higher in Steps 2 and 3 (Table 1). Therefore, AR pattern and slow speed could be useful physical markers for Step 1 and Steps 2/3, respectively. Moreover, a difference (△) of slow speed could be a marker for cryo-damage given that it is the most sensitive parameter during the sperm cryopreservation process.

The results of the current study suggest that the most critical process of cryopreservation was Step 3 and the most sensitive parameter for cryo-damage was slow speed. Moreover, we provide a comprehensive set of physical parameters for understanding cryo-damage at different steps during cryopreservation, as well as useful information to improve current preservation methods.

## Supporting Information

S1 Table Pearson correlation coeffcients among sperm parameters between Control and Step 1. (DOCX)

S2 Table Pearson correlation coeffcients among sperm parameters between Step 1 and Step 2. (DOCX)

S3 Table Pearson correlation coeffcients among sperm parameters between Step 2 and Step 3. (DOCX)

S4 Table Pearson correlation coeffcients among sperm parameters between Control and Step 3. (DOCX)

## References

[1] Olaciregui M, Gil L, Monton A, Luno V, Jerez RA, Marti JI. Cryopreservation of epididymal stallion sperm. Cryobiology. 2014;68(1):91–5. 10.1016/j.cryobiol.2013.12.009 . 24412395

[2] Mota Filho AC, Silva HV, Nunes TG, de Souza MB, de Freitas LA, de Araujo AA, et al Cryopreservation of canine epididymal sperm using ACP-106c and TRIS. Cryobiology. 2014;69(1):17–21. 10.1016/j.cryobiol.2014.04.013 . 24824725

[3] Watson PF. The causes of reduced fertility with cryopreserved semen. Animal reproduction science. 2000;60–61:481–92. . 10844218 10.1016/s0378-4320(00)00099-3

[4] Ardon F, Suarez SS. Cryopreservation increases coating of bull sperm by seminal plasma binder of sperm proteins BSP1, BSP3, and BSP5. Reproduction. 2013;146(2):111–7. 10.1530/REP-12-0468 . 23740081

[5] Brugnon F, Ouchchane L, Pons-Rejraji H, Artonne C, Farigoule M, Janny L. Density gradient centrifugation prior to cryopreservation and hypotaurine supplementation improve post-thaw quality of sperm from infertile men with oligoasthenoteratozoospermia. Human reproduction. 2013;28(8):2045–57. 10.1093/humrep/det253 . 23760160

[6] Ramon M, Perez-Guzman MD, Jimenez-Rabadan P, Esteso MC, Garcia-Alvarez O, Maroto-Morales A, et al Sperm cell population dynamics in ram semen during the cryopreservation process. PloS one. 2013;8(3):e59189 10.1371/journal.pone.0059189 23544054 PMC3609831

[7] Martins M, Justino RC, Sant'anna MC, Trautwein LG, Souza FF. Comparison of two different extenders for cryopreservation of epididymal dog sperm. Reproduction in domestic animals = Zuchthygiene. 2012;47 Suppl 6:293–4. 10.1111/rda.12042 . 23279522

[8] Peris SI, Bilodeau JF, Dufour M, Bailey JL. Impact of cryopreservation and reactive oxygen species on DNA integrity, lipid peroxidation, and functional parameters in ram sperm. Molecular reproduction and development. 2007;74(7):878–92. 10.1002/mrd.20686 . 17186553

[9] Hammerstedt RH, Graham JK, Nolan JP. Cryopreservation of mammalian sperm: what we ask them to survive. Journal of andrology. 1990;11(1):73–88. . 2179184

[10] Liu Q, Wang X, Wang W, Zhang X, Xu S, Ma D, et al Effect of the addition of six antioxidants on sperm motility, membrane integrity and mitochondrial function in red seabream (Pagrus major) sperm cryopreservation. Fish physiology and biochemistry. 2014 10.1007/s10695-014-9993-9 . 25255938

[11] Viveiros AT, Isau ZA, Caneppele D, Leal MC. Sperm cryopreservation affects postthaw motility, but not embryogenesis or larval growth in the Brazilian fish Brycon insignis (Characiformes). Theriogenology. 2012;78(4):803–10. 10.1016/j.theriogenology.2012.03.028 . 22541324

[12] Celeghini EC, de Arruda RP, de Andrade AF, Nascimento J, Raphael CF, Rodrigues PH. Effects that bovine sperm cryopreservation using two different extenders has on sperm membranes and chromatin. Animal reproduction science. 2008;104(2–4):119–31. 10.1016/j.anireprosci.2007.02.001 . 17368970

[13] Wang AW, Zhang H, Ikemoto I, Anderson DJ, Loughlin KR. Reactive oxygen species generation by seminal cells during cryopreservation. Urology. 1997;49(6):921–5. . 9187701 10.1016/s0090-4295(97)00070-8

[14] Sorrenti G, Bagnoli A, Miraglia V, Crocetta F, Vitiello V, Ristoratore F, et al Investigating sperm cryopreservation in a model tunicate, Ciona intestinalis sp. A. Cryobiology. 2014;68(1):43–9. 10.1016/j.cryobiol.2013.11.005 . 24269530

[15] D'Amours O, Frenette G, Fortier M, Leclerc P, Sullivan R. Proteomic comparison of detergent-extracted sperm proteins from bulls with different fertility indexes. Reproduction. 2010;139(3):545–56. 10.1530/REP-09-0375 . 19952166

[16] Ahmad M, Nasrullah R, Riaz H, Sattar A, Ahmad N. Changes in motility, morphology, plasma membrane and acrosome integrity during stages of cryopreservation of buck sperm. Journal of the South African Veterinary Association. 2014;85(1):972 10.4102/jsava.v85i1.972 . 24832216

[17] Wang S, Wang W, Xu Y, Tang M, Fang J, Sun H, et al Proteomic characteristics of human sperm cryopreservation. Proteomics. 2014;14(2–3):298–310. 10.1002/pmic.201300225 . 24259508

[18] Drobnis EZ, Crowe LM, Berger T, Anchordoguy TJ, Overstreet JW, Crowe JH. Cold shock damage is due to lipid phase transitions in cell membranes: a demonstration using sperm as a model. The Journal of experimental zoology. 1993;265(4):432–7. 10.1002/jez.1402650413 . 8463792

[19] Xu X, Liu Y, Cui Z, Wei Y, Zhang L. Effects of osmotic and cold shock on adherent human mesenchymal stem cells during cryopreservation. Journal of biotechnology. 2012;162(2–3):224–31. 10.1016/j.jbiotec.2012.09.004 . 22989486

[20] Ebertz SL, McGann LE. Osmotic parameters of cells from a bioengineered human corneal equivalent and consequences for cryopreservation. Cryobiology. 2002;45(2):109–17. . 12482376 10.1016/s0011-2240(02)00116-5

[21] Gilmore JA, Du J, Tao J, Peter AT, Critser JK. Osmotic properties of boar spermatozoa and their relevance to cryopreservation. Journal of reproduction and fertility. 1996;107(1):87–95. . 8699439 10.1530/jrf.0.1070087

[22] D'Amours O, Frenette G, Bordeleau LJ, Allard N, Leclerc P, Blondin P, et al Epididymosomes transfer epididymal sperm binding protein 1 (ELSPBP1) to dead spermatozoa during epididymal transit in bovine. Biology of reproduction. 2012;87(4):94 10.1095/biolreprod.112.100990 . 22875906

[23] Hinton BT, Dott HM, Setchell BP. Measurement of the motility of rat spermatozoa collected by micropuncture from the testis and from different regions along the epididymis. Journal of reproduction and fertility. 1979;55(1):167–72. . 423154 10.1530/jrf.0.0550167

[24] Pholpramool C, Zupp JL, Setchell BP. Motility of undiluted bull epididymal spermatozoa collected by micropuncture. Journal of reproduction and fertility. 1985;75(2):413–20. . 4067922 10.1530/jrf.0.0750413

[25] Shabanowitz RB, Killian GJ. Two-dimensional electrophoresis of proteins in principal cells, spermatozoa, and fluid associated with the rat epididymis. Biology of reproduction. 1987;36(3):753–68. . 3593846 10.1095/biolreprod36.3.753

[26] Awad MM, Graham JK. A new pellet technique for cryopreserving ram and bull spermatozoa using the cold surface of cattle fat. Animal reproduction science. 2004;84(1–2):83–92. 10.1016/j.anireprosci.2003.12.001 WOS:000223569500007. 15302389

[27] Kwon WS, Rahman MS, Lee JS, Kim J, Yoon SJ, Park YJ, et al A comprehensive proteomic approach to identifying capacitation related proteins in boar spermatozoa. BMC genomics. 2014;15(1):897 10.1186/1471-2164-15-897 . 25315394 PMC4287242

[28] Rahman MS, Kwon WS, Lee JS, Kim J, Yoon SJ, Park YJ, et al Sodium nitroprusside suppresses male fertility in vitro. Andrology. 2014;2(6):899–909. 10.1111/j.2047-2927.2014.00273.x . 25180787

[29] Kwon WS, Park YJ, Mohamed el SA, Pang MG. Voltage-dependent anion channels are a key factor of male fertility. Fertility and sterility. 2013;99(2):354–61. 10.1016/j.fertnstert.2012.09.021 . 23062735

[30] Ludovico P, Sansonetty F, Corte-Real M. Assessment of mitochondrial membrane potential in yeast cell populations by flow cytometry. Microbiology. 2001;147(Pt 12):3335–43. . 11739765 10.1099/00221287-147-12-3335

[31] Li JJ, Tang Q, Li Y, Hu BR, Ming ZY, Fu Q, et al Role of oxidative stress in the apoptosis of hepatocellular carcinoma induced by combination of arsenic trioxide and ascorbic acid. Acta pharmacologica Sinica. 2006;27(8):1078–84. 10.1111/j.1745-7254.2006.00345.x . 16867262

[32] Barbas JP, Mascarenhas RD. Cryopreservation of domestic animal sperm cells. Cell and tissue banking. 2009;10(1):49–62. 10.1007/s10561-008-9081-4 . 18548333

[33] Sariozkan S, Bucak MN, Tuncer PB, Ulutas PA, Bilgen A. The influence of cysteine and taurine on microscopic-oxidative stress parameters and fertilizing ability of bull semen following cryopreservation. Cryobiology. 2009;58(2):134–8. 10.1016/j.cryobiol.2008.11.006 . 19070613

[34] Kwon WS, Rahman MS, Pang MG. Diagnosis and prognosis of male infertility in mammal: the focusing of tyrosine phosphorylation and phosphotyrosine proteins. Journal of proteome research. 2014;13(11):4505–17. 10.1021/pr500524p . 25223855

[35] Rahman MS, Lee JS, Kwon WS, Pang MG. Sperm proteomics: road to male fertility and contraception. International journal of endocrinology. 2013;2013:360986 10.1155/2013/360986 24363670 PMC3864079

[36] Jin J, Jin N, Zheng H, Ro S, Tafolla D, Sanders KM, et al Catsper3 and Catsper4 are essential for sperm hyperactivated motility and male fertility in the mouse. Biology of reproduction. 2007;77(1):37–44. 10.1095/biolreprod.107.060186 . 17344468

[37] Mostafapor S, Farrokhi Ardebili F. Effects of diluting medium and holding time on sperm motility analysis by CASA in ram. Veterinary research forum: an international quarterly journal. 2014;5(2):101–5. 25568702 PMC4279634

[38] de Lamirande E, Gagnon C. Reactive oxygen species and human spermatozoa. I. Effects on the motility of intact spermatozoa and on sperm axonemes. Journal of andrology. 1992;13(5):368–78. . 1331006

[39] Kwon WS, Rahman MS, Lee JS, Kim J, Yoon SJ, Park YJ, et al A comprehensive proteomic approach to identifying capacitation related proteins in boar spermatozoa. BMC genomics. 2014;15:897 10.1186/1471-2164-15-897 25315394 PMC4287242

[40] Dona G, Kozuh I, Brunati AM, Andrisani A, Ambrosini G, Bonanni G, et al Effect of astaxanthin on human sperm capacitation. Marine drugs. 2013;11(6):1909–19. 10.3390/md11061909 23736766 PMC3721213

[41] Zaneveld LJ, De Jonge CJ, Anderson RA, Mack SR. Human sperm capacitation and the acrosome reaction. Human reproduction. 1991;6(9):1265–74. . 1752929 10.1093/oxfordjournals.humrep.a137524

[42] Garcia-Herreros M, Leal CL. Sperm volumetric dynamics during in vitro capacitation process in bovine spermatozoa. Animal: an international journal of animal bioscience. 2015:1–9. 10.1017/S1751731115000129 . 25684453

[43] Pons-Rejraji H, Bailey JL, Leclerc P. Modulation of bovine sperm signalling pathways: correlation between intracellular parameters and sperm capacitation and acrosome exocytosis. Reproduction, fertility, and development. 2009;21(4):511–24. 10.1071/RD07169 . 19383258

[44] White IG. Lipids and calcium uptake of sperm in relation to cold shock and preservation: a review. Reproduction, fertility, and development. 1993;5(6):639–58. . 9627725 10.1071/rd9930639

[45] Liu Z, Foote RH. Osmotic effects on volume and motility of bull sperm exposed to membrane permeable and nonpermeable agents. Cryobiology. 1998;37(3):207–18. 10.1006/cryo.1998.2116 . 9787066

[46] Melville DF, Johnston SD, Miller RR Jr. Flying-fox (Pteropus spp.) sperm membrane fatty acid composition, its relationship to cold shock injury and implications for cryopreservation success. Cryobiology. 2012;65(3):224–9. 10.1016/j.cryobiol.2012.06.007 . 22771758

[47] Miller RR Jr, Sheffer CJ, Cornett CL, McClean R, MacCallum C, Johnston SD. Sperm membrane fatty acid composition in the Eastern grey kangaroo (Macropus giganteus), koala (Phascolarctos cinereus), and common wombat (Vombatus ursinus) and its relationship to cold shock injury and cryopreservation success. Cryobiology. 2004;49(2):137–48. 10.1016/j.cryobiol.2004.06.002 . 15351685

[48] Kim YH, Park YJ, Yoon SJ, Kwon WS, Kim SH, Pang MG. Effect of BTS and Androhep during Storage Times on the Kinematics and Capacitation Status in Liquid Boar Semen. Reprod Dev Biol. 2010;34(3):241–6.

[49] Spungin B, Margalit I, Breitbart H. Sperm exocytosis reconstructed in a cell-free system: evidence for the involvement of phospholipase C and actin filaments in membrane fusion. Journal of cell science. 1995;108 (Pt 6):2525–35. . 7673366 10.1242/jcs.108.6.2525

[50] Kutluyer F, Kayim M, Ogretmen F, Buyukleblebici S, Tuncer PB. Cryopreservation of rainbow trout Oncorhynchus mykiss spermatozoa: effects of extender supplemented with different antioxidants on sperm motility, velocity and fertility. Cryobiology. 2014;69(3):462–6. 10.1016/j.cryobiol.2014.10.005 . 25445462

[51] Alapati R, Stout M, Saenz J, Gentry GT Jr, Godke RA, Devireddy RV. Comparison of the permeability properties and post-thaw motility of ejaculated and epididymal bovine spermatozoa. Cryobiology. 2009;59(2):164–70. 10.1016/j.cryobiol.2009.06.009 . 19545558

[52] Lessard C, Parent S, Leclerc P, Bailey JL, Sullivan R. Cryopreservation alters the levels of the bull sperm surface protein P25b. Journal of andrology. 2000;21(5):700–7. . 10975417

[53] Prathalingam NS, Holt WV, Revell SG, Mirczuk S, Fleck RA, Watson PF. Impact of antifreeze proteins and antifreeze glycoproteins on bovine sperm during freeze-thaw. Theriogenology. 2006;66(8):1894–900. 10.1016/j.theriogenology.2006.04.041 . 16777208

[54] Ball BA, Vo A. Osmotic tolerance of equine spermatozoa and the effects of soluble cryoprotectants on equine sperm motility, viability, and mitochondrial membrane potential. Journal of andrology. 2001;22(6):1061–9. . 11700853 10.1002/j.1939-4640.2001.tb03446.x

[55] De Leeuw FE, Chen HC, Colenbrander B, Verkleij AJ. Cold-induced ultrastructural changes in bull and boar sperm plasma membranes. Cryobiology. 1990;27(2):171–83. . 2331890 10.1016/0011-2240(90)90009-s

