# Supplementary material for: A Novel Approach to Identifying Physical Markers of Cryo-Damage in Bull Spermatozoa
Source: PLoS One. 2015 May 4;10(5):e0126232. doi: 10.1371/journal.pone.0126232 (PMC4418755; doi:10.1371/journal.pone.0126232)
Supplement: S3 Table — (DOCX) [file pone.0126232.s003.docx]

**Supporting Information**


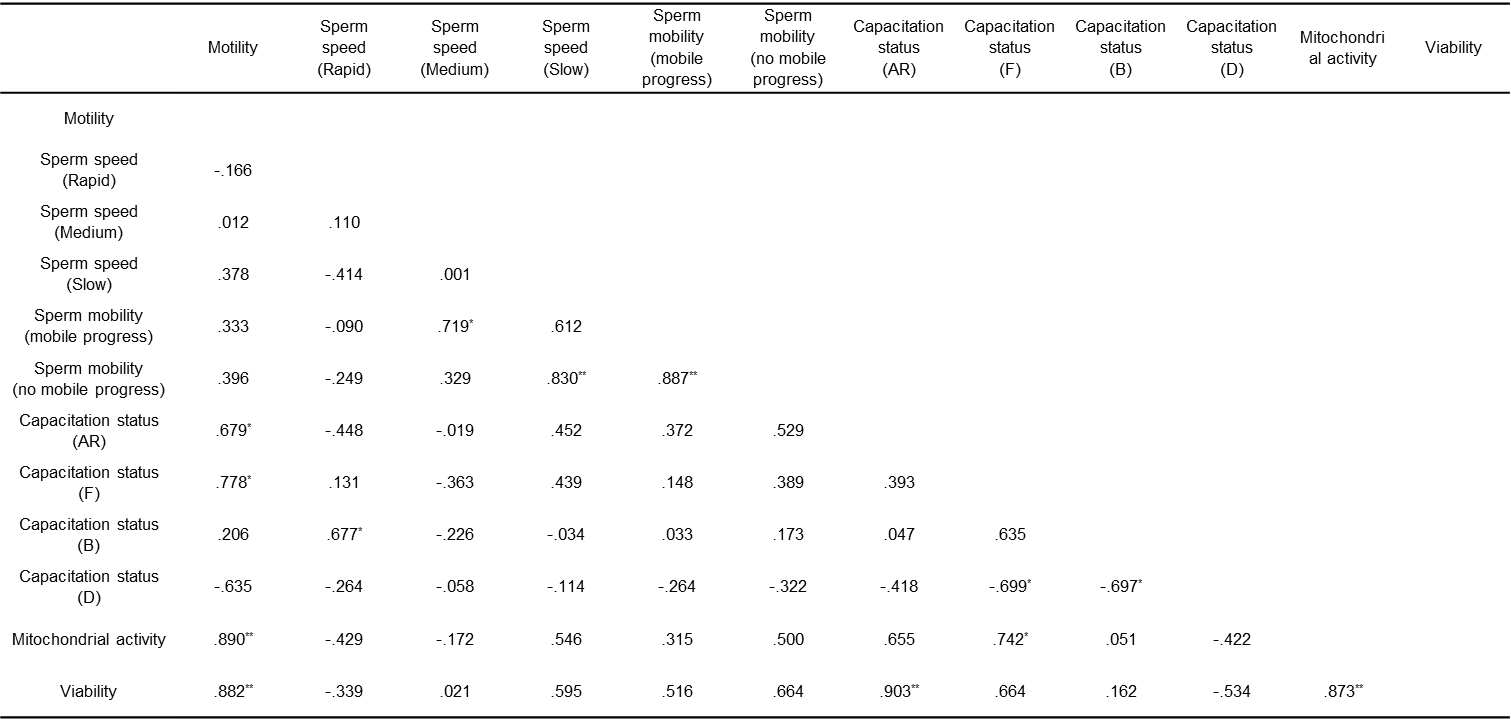
Table S3. Pearson correlation coeffcients among sperm parameters between Step 2 and Step 3

* P < 0.05; ** P < 0.01
